# Plantain peel extract-mediated synthesis of CuO nanoparticles: comprehensive characterization, bioinertness *in vitro* and *in vivo*, and anticancer evaluation

**DOI:** 10.5599/admet.3203

**Published:** 2026-03-24

**Authors:** Srimathi JaganMoorthy, Pranav Raaj Subbarayan Ravichandar, Harini Ganesan, Balasubramanian Deepika, Pazhani Durgadevi, Arulsamy Arokyapraveen, Agnishwar Girigoswami, Koyeli Girigoswami

**Affiliations:** 1 Saveetha Medical College and Hospital, Saveetha Institute of Medical and Technical Sciences, Thandalam, Chennai, 602105, India; 2 Department of Pathology, Saveetha Medical College and Hospital, Saveetha Institute of Medical and Technical Sciences, Thandalam, Chennai, 602105, India; 3 Faculty of Allied Health Sciences, Chettinad Hospital and Research Institute, Chettinad Academy of Research and Education, Chettinad Health City, Kelambakkam, 603103, Tamilnadu, India; 4 Medical Bionanotechnology Lab, Department of Obstetrics and Gynaecology, Saveetha Medical College and Hospital, Saveetha Institute of Medical and Technical Sciences, Thandalam, Chennai, 602105, India

**Keywords:** Health care, copper oxide nanoparticles, HepG2 cell killing, zebrafish embryos, plantain peel

## Abstract

**Background and purpose:**

Cancer is one of the leading causes of death worldwide, failing to identify a complete cure. Liver cancer is the sixth most frequent cancer worldwide, and its cure is still not assured. Nanoparticles, especially the metal oxide nanoparticles, have been explored as anticancer agents in recent times. In an attempt to make the best use of waste, plantain peel, a byproduct of agriculture, was used to synthesize copper oxide nanoparticles.

**Experimental approach:**

The plantain peel extract was evaluated for its chemical composition by GC-MS analysis, which revealed a predominant presence of tetratetracontane. The copper oxide nanoparticles were characterized by UV-visible spectrophotometry, dynamic light scattering, zeta potential, Fourier transform infrared spectroscopy (FTIR), X-ray diffraction (XRD), scanning electron microscopy (SEM) and energy-dispersive X-ray spectroscopy (EDAX). Bio-inertness was assessed using an MTT assay of fibroblasts, a haemolysis assay, and zebrafish embryo analysis. The anticancer activity against HepG2 cells was estimated.

**Key results:**

The absorption peak was found at 402 nm, the hydrodynamic diameter was 323 nm, the zeta potential was +10.74 mV, and a band gap of 1.3 eV. The SEM images showed a size range of 54 to 85 nm with a chrysanthemum-petal-like morphology. EDAX showed the presence of Cu and O, and the XRD and FTIR peaks corroborated with that of CuO. The result showed that up to a dose of 50 μg mL^-1^, the nanoparticles did not induce any toxicity. Finally, the anticancer activity, evaluated using the HepG2 cell line, showed a dose-dependent cytotoxic effect with an IC50 of 26.20 μg mL^-1^.

**Conclusion:**

The outcome of this study suggested that the synthesized copper oxide nanoparticles can be used at controlled doses to kill cancer cells. Further studies are needed using other cancer cell lines and *in vivo* cancer models.

## Introduction

Cancer comprises a group of complex diseases marked by uncontrolled cell growth, abnormal proliferation, tissue invasion, and metastasis. It develops due to changes in genetic and epigenetic makeup, which can disrupt normal regulation of cell division, differentiation, and apoptosis induction [1]. These changes may arise from inherited mutations or external factors such as carcinogens, radiation, oncogenic viruses, and lifestyle influences. Cancer progression occurs through initiation, promotion, and malignant transformation, leading to tumour formation and systemic spread. The disease is highly heterogeneous, differing in molecular characteristics, aggressiveness, and treatment response. Despite advances in diagnosis and therapies, cancer remains a major global health burden [2,3]. Apart from conventional therapies such as chemotherapy, radiation, surgical removal of the tumour, immunotherapy, and targeted therapy, nanoparticles have also been shown to enhance treatment efficacy [4-6].

Nanotechnology is a rapidly growing field with numerous applications across industries, including environmental sciences [7], medicine [8,9], food [10], and health [11,12]. Metals and metal oxides constitute one of the most diverse classes of materials in chemistry, as their unique properties span almost every domain of science and technology. Among them, transition metal oxides are particularly significant due to their advanced technological relevance and economic importance. Copper oxide nanoparticles (CuO NPs) have attracted considerable attention due to their ease of synthesis, high stability, and cost-effectiveness compared with noble metals such as gold and silver [13,14]. In addition, CuO nanoparticles exhibit stability over a broad pH range and are highly resistant to high temperatures [15-17]. Recently, CuO NPs have garnered significant interest, mainly due to their antimicrobial and biocidal properties, and they are used extensively across various applications in the healthcare sector [18-20].

Chemical synthesis of any nanoparticle poses a threat to the environment as well as living beings, including humans. Consequently, the adoption of green synthesis techniques for CuO NPs, which are environmentally friendly and sustainable, has gained significant traction as a replacement for traditional synthesis methods [21]. In green synthesis, plant-mediated products are more likely to serve as reducing agents. Various metabolites and functional groups present in the biological products play important roles in reducing, chelating, stabilizing, and synthesizing nanoparticles [22]. Nowadays, fruit peels are widely recognized as natural sources of antioxidants and phytochemicals with strong free-radical–scavenging activity. These bioactive compounds play a key role in reducing metal salts to their metallic form, which are subsequently oxidized in air to yield metal oxide nanoparticles. Plantain peels, in particular, represent a major agricultural by-product generated from food waste, as the peels are typically discarded after consumption of the edible pulp. In addition to their abundance, plantain peels have also been traditionally utilized in wound healing and medicinal applications [23,24].

In the present study, CuO NPs were synthesized using plantain peel and characterized using UV-vis spectroscopy, Fourier transform infrared spectroscopy (FTIR), X-ray diffraction (XRD), dynamic light scattering (DLS), zeta potential, energy-dispersive X-ray spectroscopy (EDAX) and scanning electron microscopy (SEM) to evaluate their structural, morphological, and optical properties. The components of plantain peel extract were estimated using phytochemical analysis and GC-MS analysis. The biological compatibility was estimated using the MTT assay and zebrafish embryo study. Finally, the anticancer activity of the as-synthesized BP-CuO nanoparticles was evaluated using HepG2 (hepatocellular carcinoma) cells.

## Experimental

### Materials

Plantain was purchased from the local market at Kelambakkam, Tamilnadu, India. Copper chloride (CuCl_2_), sodium hydroxide (NaOH), Potassium bromide (KBr), Dulbecco’s minimal essential medium (DMEM), phosphate buffer saline (PBS) tablets of pH 7.4, antibiotic-antimycotic solution, 3-(4,5-dimethylthiazol-2-yl)-2,5--diphenyltetrazolium bromide (MTT), dimethyl sulfoxide (DMSO), 2,2-diphenyl-1-picrylhydrazyl (DPPH), trypsin-EDTA, and other chemicals were purchased from HiMedia Pvt. Ltd, India. Fetal bovine serum was purchased from Gibco, USA. V79 and HepG2 cells were ordered from Curator, NCCS, Pune, India.

### Plantain peel extract preparation

Plantain (*Musa paradisiaca*) peels were collected, thoroughly washed with distilled water to remove dirt and impurities, and then air-dried. A measured amount of 0.25 g of finely ground plantain peel powder is taken and mixed with an appropriate volume (100 mL) of distilled water (D/W). The mixture was initially dissolved by magnetic stirring for 2 h at 70 °C and followed by ultrasonication at 50 Hz for 15 minutes (repeated 4 times) to ensure maximum extraction of bioactive compounds. After ultrasonication, the solution was subjected to magnetic stirring at 500 rpm for 1 hour to promote uniform extraction. The extract was later filtered through a Whatman filter paper to remove solid residues and to yield a clear aqueous plantain peel extract. The overall yield of the extract was approximately 20 to 25 %. The entire experiment was conducted using the same batch of extract.

### Phytochemical analysis and GC-MS of the plantain peel extract

Phytochemical analysis of the plantain peel (or raw banana peel, BP) extract was performed to identify the phytochemicals present in it, according to Garg et al. [25]. The aqueous extract of plantain peel was completely dried and redissolved in methanol for GC-MS analysis. The GC-MS analysis was performed using PerkinElmer Clarus 680 & 600. GC-MS instrument as done earlier [7]. The identification of the compounds was based on retention time, fragmentation patterns, and automated mass spectral deconvolution and identification software using NIST.

### Synthesis

CuCl_2_ (0.1 mol L^-1^) was dissolved in plantain peel extract and added dropwise to the appropriate amount (50 mL) of aqueous NaOH (0.1 mol L^-1^) under constant stirring, leading to anion-cation interaction and product formation. After the reaction was complete, the product was allowed to precipitate overnight, and the mother liquid was discarded, retaining the precipitate in the beaker. The precipitate thus obtained was washed three times using D/W by centrifuging at 5000 rpm each time. The pellet obtained was dried in a porcelain crucible and calcined at 200 °C for 3 h. The powder was crushed using a mortar and pestle, and the ground powder was BP-CuO nanoparticles.

### Characterization

The characterization of BP-CuO nanoparticles involved various analytical techniques to evaluate their structural, morphological, functional, and stability-related properties. Ultraviolet-visible (UV-vis) spectroscopy was used to confirm nanoparticle formation by characterizing surface plasmon resonance (SPR) absorption peaks. For UV-visible spectrophotometry, DLS and zeta potential measurements, the nanoparticle solution was diluted with 0.9 % NaCl (normal saline) and sonicated for 15 min using an ultrasonicator to prevent aggregation. DLS provides data on the size distribution and hydrodynamic diameter of a particle in a colloidal suspension, and zeta potential analysis informs us about the surface charge and colloidal stability of the nanoparticles in aqueous environments. Malvern ZetaSizer was used to measure DLS and zeta potential, whereas JASCO UV-Vis V-730 was used to measure UV-vis absorption spectra.

Further, the dried powder of the synthesized nanoparticles was subjected to X-ray diffraction (XRD) analysis (Unique D8 diffractometer) to determine the crystalline structure, phase purity, and average crystallite size. Scanning electron microscopy (SEM) (FEI –TECNAI, G2-20 TWIN) was used to examine the surface morphology, particle size distribution, and offer insights into the shape and agglomeration tendencies of the nanoparticles. For SEM analysis, the powdered samples were uniformly coated with a conducting carbon tape, followed by gold and palladium coatings for improving its conductivity. EDAX analysis was done to determine the composition of the nanoparticles. Fourier transform infrared spectroscopy was conducted through the KBr pellet method using a Bruker Alpha FTIR spectrometer, and the spectrum was recorded through transmittance mode to identify the functional groups present in the BP-CuO nanoparticles.

### Antioxidant assay

The antioxidant activity of the as-synthesized BP-CuO nanoparticles was estimated using the 2,2-diphenyl--1-picrylhydrazyl (DPPH) assay. Ascorbic acid was used as the standard, and the procedure followed was the same as that used earlier [7].

### Biological interaction and toxicity assessment

#### MTT assay using normal cell lines

Chinese hamster lung fibroblast (V79) cell lines were used to assess the effect of BP-CuO nanoparticles on normal cells. This aspect is very important because any agent used for anticancer activity should not affect normal cells. They should specifically kill the cancer cells. V79 cells were grown in complete medium (DMEM containing 10 % FBS, 1 % antibiotics) and seeded in a 48-well plate. After 24 h, the cells were treated with BP-CuO nanoparticles suspended in complete medium and filtered using a sterile 0.22 μm filter. The doses tested were 10, 25, 50 and 100 μg mL^-1^ and the samples were further incubated for an additional 24 h; inverted microscopic images were captured. MTT was added, and the plates were incubated in the dark in a CO_2_ incubator for 4 h. After the formazan crystals were formed, they were dissolved in DMSO, and the optical density was recorded at 570 nm. The percentage of cell viability was calculated as done earlier [26].

#### Haemolysis assay

The haemolysis assay was performed to assess the blood compatibility of the synthesized BP-CuO nanoparticles with human blood. The protocol was conducted in accordance with the method reported in our previous study [27]. Briefly, 3 mL of blood was obtained from a healthy 21-year-old male volunteer using an EDTA-coated vacutainer after obtaining informed consent. The appropriate permission was obtained from the Institutional Ethics Committee of Saveetha Medical College and Hospital, SIMATS, Thandalam, Chennai, India. (005/07/2025/IEC/SMCH), dated 04.08.2025. The Declaration of Helsinki was followed strictly. The blood sample was centrifuged at 1500 rpm for 15 minutes to separate the red blood cell (RBC) pellet, which was subsequently washed three times with 0.9 % normal saline. The washed RBCs were then diluted tenfold with saline and aliquoted into five tubes (in triplicate; this means a total of 15 tubes), each containing 100 μL of RBC suspension and 900 μL of BP-CuO nanoparticle suspensions prepared in normal saline at different concentrations (10, 25 and 50 μg mL^-1^). D/W was utilized as the positive control to induce complete haemolysis, while normal saline served as the negative control. All samples were incubated at 37 °C for 2 h and then centrifuged at 12,000 rpm for 1 minute. After centrifugation, photographic documentation was performed. The supernatants were carefully collected into fresh tubes, and the absorbance was measured at 541 nm, corresponding to the maximum absorbance of haemoglobin. The percentage of haemolysis was calculated using the formula previously published [27].

#### *In vivo* toxicity assessment using zebrafish embryos

Transparent zebrafish embryos (8 h post-fertilization) were collected and distributed into a 24-well plate, with 10 embryos per well. In triplicates the embryos were treated with BP-CuO nanoparticles at 10, 25 and 50, and images of the developing embryos were captured using a light microscope. The embryo hatching was noted, and cumulative hatchability was calculated and plotted.

### Anticancer activity in vitro using liver cancer cell lines

The HepG2 (liver cancer) cell line was used to assess the anticancer activity. MTT assay was done after treatment with BP-CuO nanoparticles, and the cell viability percentage was estimated as mentioned in the section “*MTT assay using normal cell lines*”.

### Statistical analysis

Statistical significance level was calculated based on the unpaired Student’s t-test. The results are repeated in triplicate and the data are represented as mean ± standard error of the mean, *p* < 0.001, which is taken as significant compared to the control.

## Results and discussion

### Composition of plantain peel extract

**Figure 1.**
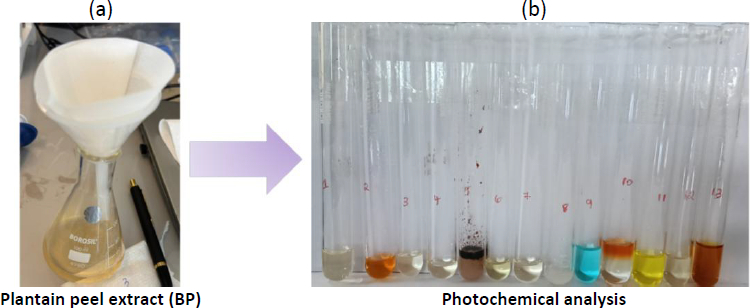
(a) The extraction of plantain peel and filtration, (b) phytochemical analysis of plantain peel extract

Figure 1a shows the filtration of the plantain peel extract; Figure 1b shows the phytochemical analysis of plantain peel extract, detecting the presence of alkaloids, phenols, tannins, reducing sugars, proteins, and saponins, and Figure S1 (Supplementary material) shows the GC-MS profile of the plantain peel containing the following chemicals, as shown in Table 1.

**Table 1.** The chemical components available in the plantain peel extract, obtained by GC-MS analysis

No.	Compound Name	Molecular formula	Molecular weight	Abundance, %
1	Octadecanoic acid, phenylmethyl ester	C_25_H_42_O_2_	374	9.11
2	Dodecanoic acid, phenylmethyl ester	C_19_H_30_O_2_	290	8.69
3	7,7-Diethylheptadecane	C_21_H_44_	296	4.22
4	Tetratetracontane	C_44_H_90_	618	69.64
5	Propionic acid, 3-iodo-, heptadecyl ester	C_20_H_39_IO_2_	438	8.34

The NIST library matched for the plantain peel GC-MS spectra reveals the presence of octadecanoic acid, phenylmethyl ester; dodecanoic acid, phenylmethyl ester; 7,7-Diethylheptadecane; tetratetracontane; propionic acid, 3-iodo-, heptadecyl ester are shown in (Figure S2a to S2e), respectively. From the GC-MS results, tetratetracontane was found to be present at the highest abundance (69.64 %) in plantain peel. Tetratetracontane is a long-chain alkane and represents one of the principal components of the essential oils derived from *Euphorbia macroclada* [28,29]. Being a natural product, tetratetracontane has shown diverse beneficial effects, including anti-lipid peroxidation and radical-scavenging activities [30]. This can be beneficial for its use as a protective agent to mitigate the harmful effects of nanoparticles or other anticancer agents.

### BP-CuO characterization

**Figure 2.**
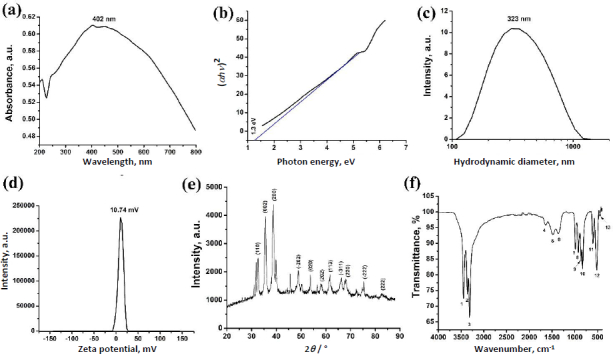
(a) absorption spectrum, (b) band gap, (c) hydrodynamic diameter, (d) zeta potential, (e) XRD spectrum and (f) FTIR spectrum of the as-synthesized BP-CuO nanoparticles

The absorption spectrum of BP-CuO nanoparticles shows a peak at 402 nm (Figure 2a), consistent with the typical absorption peak of CuO. In previous studies, a similar absorption peak for CuO nanoparticles has been reported at 400 nm [31]. The band gap calculated using Tauc’s plot (Figure 2b) was found to be 1.3 eV, which confirms a very good semiconducting property [32,33]. The hydrodynamic diameter of the BP-CuO nanoparticles was found to be 323 nm (Figure 2c), indicating nanometer-sized particles, and the stability was moderate, as evidenced by a zeta potential of +10.74 mV (Figure 2d).

The XRD data (Figure 2e) show that the Miller indices match with the previously reported XRD peaks [34] and the JCPDS card number JCPDS-45-0937 [35]. This result confirms the proper synthesis of CuO, with no other compounds present. The FTIR spectrum of BP-CuO is shown in Figure 2f, and the respective peaks are summarized in Table 2.

**Table 2.** The FTIR peaks obtained for the BP-CuO nanoparticles and their corresponding bonds

Peak No. in Fig 2f	Wavenumber, cm^-1^	Corresponding bond stretching/ vibration/ bending/ twisting/ rotation	Ref.
1	3359	O-H	[36]
2	3446	O-H	[36]
3	3313	O-H	[36]
4	1637	C=O stretching	[37]
5	1481	C=O	[38]
6	1363	alkoxy -C–O- stretching bonds	[39,40]
7	987	C=O	[41]
8	922	C=C	[36]
9	863	C-C	[42]
10	828	C-C	[42]
11	602	Cu-O	[37,43]
12	517	Cu-O	[37,43]
13	447	Cu-O	[37,43]

**Figure 3.**
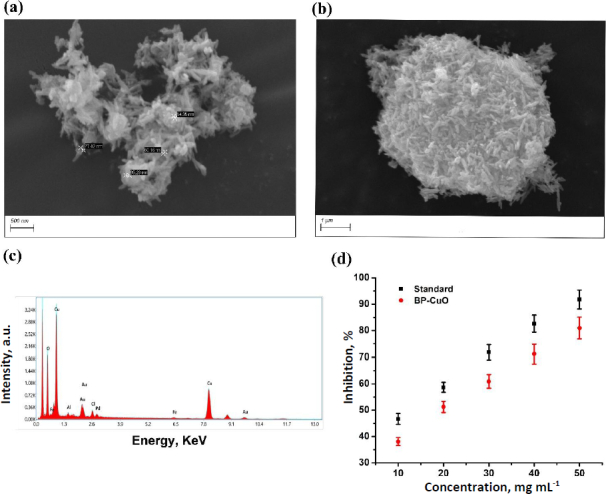
(a) and (b) The SEM images of the synthesized BP-CuO nanoparticles at different magnifications, (c) The EDAX of the synthesized BP-CuO nanoparticles and (d) antioxidant activity of the BP-CuO nanoparticles

The SEM analysis of the synthesized BP-CuO nanoparticles shows a typical rod-like structure assembled in a random orientation, giving it a look like a chrysanthemum flower (Figure 3a and 3b). The size of the petals of the flower was found in the range of 54 to 85 nm. The EDAX analysis shows the presence of Cu and O, confirming the synthesis (Figure 3c). The antioxidant activity was also evaluated by the DPPH assay, and the results shown in Figure 3d indicate that the nanoparticles exhibited some antioxidant activity as well. However, the antioxidant activity of the standard (ascorbic acid) was higher compared to the nanoparticle at every respective concentration evaluated. This may be due to the presence of tetratetracontane in the plantain peel, as revealed by GC-MS analysis.

### In vitro and in vivo biocompatible nature of BP-CuO nanoparticles

**Figure 4.**
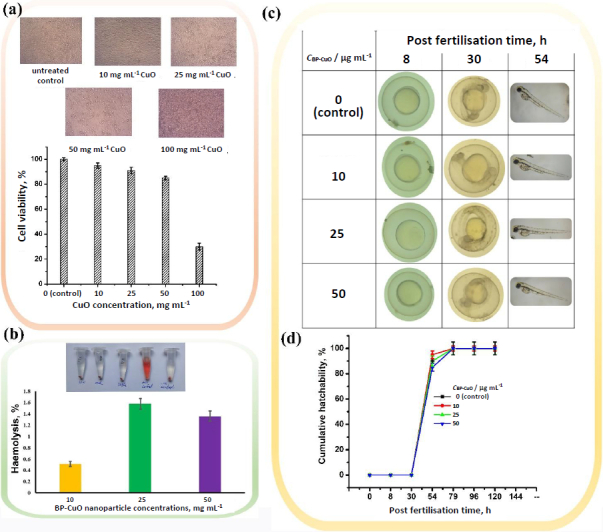
(a) Cell viability of V79 cells using the MTT assay. The inverted microscopic images are shown in the inset; (b) The haemolysis after exposure to various BP-CuO nanoparticle concentrations (inset shows the tube images after haemolysis); (c) the light microscopic image of the zebrafish embryos and (d) cumulative hatchability at different post fertilisation time, post BP-CuO nanoparticle exposure

The MTT assay data, as shown in Figure 4a shows that the nanoparticles (BP-CuO) were not toxic up to a dose of 50 μg mL^-1^. The cell morphology was also bipolar, and the cells appeared healthy. However, at a 100 μg mL^-1^ dose, the cells’ morphology became round, and they began to float, which is a hallmark of dead cells. Cell viability decreased to 30 ± 2.7 % after exposure to 100 μg mL^-1^ BP-CuO. Thus, the nanoparticles were benign and could be used up to a dose of 50 μg mL^-1^, at which normal cell viability was 85 ± 1.4 %. On the other hand, the haemolysis assay data (Figure 4b) showed that the percentage of haemolysis remained below 2 % up to a dose of 50 μg mL^-1^. It has been reported that haemolysis of up to 5 % can be considered safe for human use [44-46].

The effect of BP-CuO nanoparticles on zebrafish embryos is visible in Figure 4c. No developmental defects were observed in the treated embryos, and morphology was similar to that of the untreated control group. The cumulative hatchability data (Figure 4d) also showed that the nanoparticles did not cause any death or delayed hatching up to a dose of 50 μg mL^-1^, indicating that the synthesized BP-CuO nanoparticles were not toxic. An earlier report shows that green- and chemically-synthesized CuO nanoparticles elicited developmental toxicity at a 200 μg mL^-1^ dose [47]. Tail bend, axis bend, yolk sac oedema, tail fold malformation, and head malformation were observed by Sabeena *et al.* [48] after exposure to 100 μL of synthesized CuO nanoparticles, using the leaf extract of *Salacia reticulate*, a medicinal plant. Thus, our findings are consistent with earlier reports, and 50 μg mL^-1^ is benign for the embryos.

### Anticancer activity assessment

**Figure 5.**
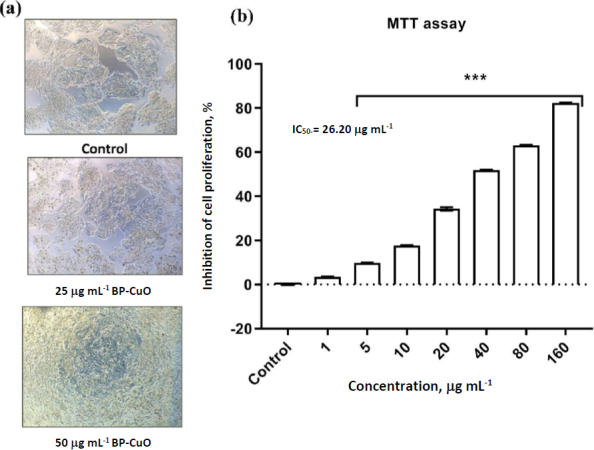
(a) The inverted microscopic image and (b) cell viability of HepG2 cells after exposure to different doses of BP-CuO nanoparticles.

HepG2 cells are a widely used human liver cancer cell line derived from hepatocellular carcinoma. They retain many hepatocyte-like functions, including protein synthesis, lipid metabolism, and detoxification pathways, making them valuable for studies on liver physiology, drug toxicity, and metabolic regulation [49]. The cancer cell-killing capacity of BP-CuO nanoparticles was estimated, and the results showed a dose-dependent effect (Figure 5b). The inverted microscopic images (Figure 5a) showed a decrease in cell density with increasing exposure dose. The IC_50_ value was estimated to be 26.20 μg mL^-1^.

In a previous study, HepG2 cells were treated with nano-Cu, nano-CuO, or CuCl_2_ ions. Exposure to nano-Cu and nano-CuO produced limited signs of cytotoxicity within the concentration range of 0.1 to 3 μg mL^-1^. Among the dose-dependent responses, lactate dehydrogenase and aspartate transaminase were the most sensitive indicators of cytotoxicity. Alanine aminotransferase, glutathione reductase, glucose-6-phosphate dehydrogenase, and total protein content showed the next highest level of responsiveness. Moderate responses were observed for superoxide dismutase, gamma-glutamyl transpeptidase, total bilirubin, and microalbumin. Notably, glutathione peroxidase, glutathione reductase, and protein levels were altered following exposure to nano-Cu and nano-CuO, whereas no such changes were detected with CuCl_2_ treatment [50]. Younas *et al.* [51] reported that exposure to CuO_2_ nanoparticles reduced cell viability in HepG2 cells by 46 % at 100 μg mL^-1^. Thus, our results showing the cancer cell-killing effect can be used as a potential therapeutic agent in the future.

The green synthesis of copper oxide (CuO) nanoparticles using *Musa paradisiaca* (plantain peel) extract represents an eco-friendly, cost-effective, and sustainable approach for nanoparticle production. The phytochemical screening of the aqueous peel extract revealed the presence of flavonoids, tannins, saponins, alkaloids, glycosides, and reducing sugars, which act as natural reducing and stabilizing agents. These bioactive compounds facilitated the formation of CuO nanoparticles by reducing Cu^2+^ ions and stabilizing the resulting nanoparticles. The synthesized particles exhibited chrysanthemum flower morphology with petal-like protrusions, as observed under SEM, with an average size of 63 nm. DLS analysis showed a hydrodynamic size of 323 nm and a zeta potential of +10.74 mV, indicating moderate colloidal stability suitable for biological applications. Further characterization using UV-vis spectroscopy confirmed the formation of nanoparticles through surface plasmon resonance, while FTIR analysis identified functional groups responsible for bio-reduction and capping. XRD confirmed the crystalline nature of CuO, and GC-MS analysis validated the presence of secondary metabolites in the plant extract.

Biocompatibility was assessed through both *in vitro* and *in vivo* approaches. MTT and haemolysis assays demonstrated that the CuO nanoparticles exhibited dose-dependent cytocompatibility, with high cell viability at lower concentrations and haemolysis rates under 5 %, signifying excellent blood compatibility and minimal cytotoxicity. These findings suggest that these green-synthesized nanoparticles are safe for interaction with normal cells and blood components. *In vivo* toxicity was evaluated using zebrafish embryo assays, which revealed no significant developmental abnormalities, mortality, or reduction in hatchability across concentrations ranging from 10 to 50 μg mL^-1^. This supports the claim that nanoparticles possess low embryotoxicity and are safe for use in living systems at biologically relevant doses.

The anticancer potential of these biosynthesized CuO nanoparticles has been thoroughly supported by *in vitro* assays, with an IC_50_ of 26.20 μg mL^-1^. The DPPH assay confirmed the moderate antioxidant capacity of the nanoparticles, which is crucial in neutralizing free radicals and mitigating oxidative stress, a key contributor to cancer progression. The phytochemicals from the plantain peel play a pivotal role here by donating hydrogen atoms or electrons to stabilize the DPPH radical, thereby protecting cellular components from oxidative damage.

## Conclusions

These findings collectively demonstrate the efficiency and reliability of plantain peel extract for the green synthesis of CuO nanoparticles. The stable structural properties, suitable particle size, and functionalization with natural biomolecules give strong potential for the application of the nanoparticles in biomedical fields, such as antimicrobial treatments, cancer therapy, drug delivery systems, and environmental remediation catalytic processes. In future studies, it could focus on optimizing reaction parameters and evaluating the *in vivo* cancer tumour-reducing effects of the nanoparticles. It summarizes that the study not only adds value to agricultural waste, such as plantain peel, but it also contributes to the development of sustainable nanomaterials with versatile applications.

## Supplementary material

Additional data are available at https://pub.iapchem.org/ojs/index.php/admet/article/view/3203, or from the corresponding authors on request.



## Abbreviations

BP: plantain peel/ banana peel
GC-MS: Gas chromatography mass spectrometry
BP-CuO: CuO nanoparticles synthesized using raw banana (plantain) peel
UV: ultraviolet
FTIR: -Fourier transform infrared spectroscopy
XRD: X-ray diffractometry
SEM: Scanning electron microscopy
EDAX: -Energy-dispersive X-ray spectroscopy
MTT: 3-(4,5-di methyl thiazol-2-yl)-2,5-diphenyltetrazolium bromide
DLS: Dynamic light scattering
DMEM: Dulbecco's Modified Eagle Medium
DMSO: Dimethyl sulphoxide
NIST: The National Institute of Standards and Technology
FBS: Foetal bovine serum
SPR: Surface plasmon resonance
DPPH: 2,2-diphenyl-1-picrylhydrazyl
RBC: red blood cells
