# Supplementary material for: Plantain peel extract-mediated synthesis of CuO nanoparticles: comprehensive characterization, bioinertness in vitro and in vivo, and anticancer evaluation
Source: ADMET DMPK. 2026 Mar 24;14:3203. doi: 10.5599/admet.3203 (PMC13147518; doi:10.5599/admet.3203)
Supplement: Supplementary file 1 [file ADMET-14-3203-S1.pdf]

ADMET & DMPK **14** (2026) S3203

Open Access : ISSN : 1848-7718

http://www.pub.iapchem.org/ojs/index.php/admet/index

Supplementary material to

Plantain peel extract-mediated synthesis of CuO nanoparticles: comprehensive characterization, bioinertness *in vitro* and *in vivo*, and anticancer evaluation

Srimathi JaganMoorthy[1], Pranav Raaj Subbarayan Ravichandar[1], Harini Ganesan[1], Balasubramanian Deepika[2], Pazhani Durgadevi[3], Arulsamy Arokyapraveen[3], Agnishwar Girigoswami[3] and Koyeli Girigoswami[4,]

[1]*Saveetha Medical College and Hospital, Saveetha Institute of Medical and Technical Sciences, Thandalam, Chennai, 602105, India*

[2]*Department of Pathology, Saveetha Medical College and Hospital, Saveetha Institute of Medical and Technical Sciences, Thandalam, Chennai, 602105, India*

[3]*Faculty of Allied Health Sciences, Chettinad Hospital and Research Institute, Chettinad Academy of Research and Education, Chettinad Health City, Kelambakkam, 603103, Tamilnadu, India*

[4]*Medical Bionanotechnology Lab, Department of Obstetrics and Gynaecology, Saveetha Medical College and Hospital, Saveetha Institute of Medical and Technical Sciences, Thandalam, Chennai, 602105, India*

ADMET & DMPK **14** (2026) 3203; https://doi.org/10.5599/admet.3203

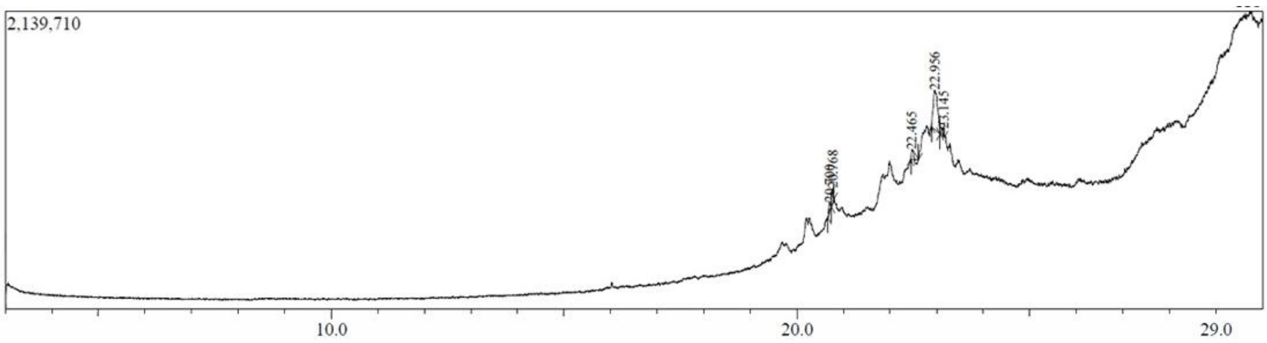

Time, min

Figure S1. GC-MS spectrum of the methanol suspended plantain peel extract

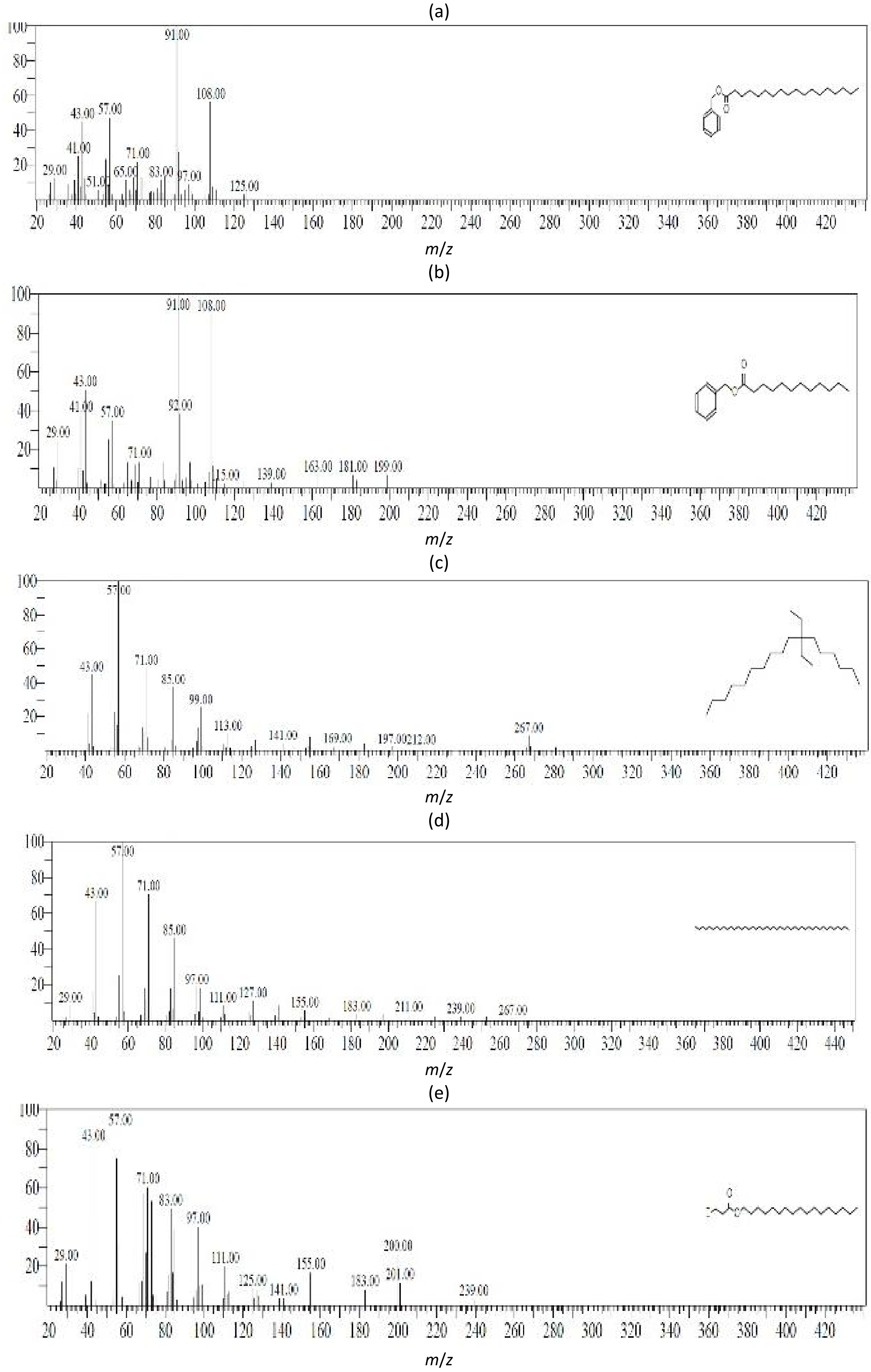

Figure S2. (a-e) The NIST library matches representing the spectra- octadecanoic acid, phenylmethyl ester; dodecanoic acid, phenylmethyl ester; 7,7-Diethylheptadecane; tetratetracontane; propionic acid, 3-iodo-, heptadecyl ester, respectively.
